# Development of an artificial intelligence-based multimodal model for assisting in the diagnosis of necrotizing enterocolitis in newborns: a retrospective study

**DOI:** 10.3389/fped.2024.1388320

**Published:** 2024-05-17

**Authors:** Kaijie Cui, Shao Changrong, Yu Maomin, Zhang Hui, Liu Xiuxiang

**Affiliations:** ^1^ Neonatal Intensive Care Unit, Women and Children’s Hospital, Qingdao University, Qingdao, China; ^2^ Department of Pediatrics, Qilu Hospital of Shandong University, Qingdao, China; ^3^ Department of Pediatrics, Qingdao Eighth People’s Hospital, Qingdao, China; ^4^ Department of Neonatology, Second Affiliated Hospital of Shandong First Medical University, Jinan, China

**Keywords:** artificial intelligence, necrotizing enterocolitis, multimodal model, attention analysis, computer-aided diagnosis

## Abstract

**Objective:**

The purpose of this study is to develop a multimodal model based on artificial intelligence to assist clinical doctors in the early diagnosis of necrotizing enterocolitis in newborns.

**Methods:**

This study is a retrospective study that collected the initial laboratory test results and abdominal x-ray image data of newborns (non-NEC, NEC) admitted to our hospital from January 2022 to January 2024.A multimodal model was developed to differentiate multimodal data, trained on the training dataset, and evaluated on the validation dataset. The interpretability was enhanced by incorporating the Gradient-weighted Class Activation Mapping (GradCAM) analysis to analyze the attention mechanism of the multimodal model, and finally compared and evaluated with clinical doctors on external datasets.

**Results:**

The dataset constructed in this study included 11,016 laboratory examination data from 408 children and 408 image data. When applied to the validation dataset, the area under the curve was 0.91, and the accuracy was 0.94. The GradCAM analysis shows that the model's attention is focused on the fixed dilatation of the intestinal folds, intestinal wall edema, interintestinal gas, and portal venous gas. External validation demonstrated that the multimodal model had comparable accuracy to pediatric doctors with ten years of clinical experience in identification.

**Conclusion:**

The multimodal model we developed can assist doctors in early and accurate diagnosis of NEC, providing a new approach for assisting diagnosis in underdeveloped medical areas.

## Graphical Abstract

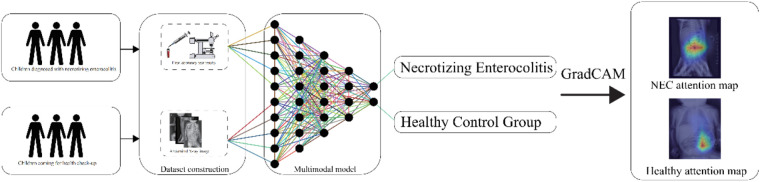

## Introduction

Necrotizing enterocolitis (NEC) is the leading cause of neonatal death (1–3). The incidence of NEC is approximately 1%–3%, with a mortality rate of 15%–30% (4). In extremely low birth weight infants, the incidence of NEC can reach 5%–16%, with a mortality rate as high as 20%–50% (5). Surviving NEC infants often suffer from severe complications such as intestinal failure, short bowel syndrome, growth retardation, and neurological developmental disorders, which greatly affect their quality of life (6–9). Early diagnosis of NEC is crucial for improving the prognosis of affected infants. Currently, the diagnosis of NEC mainly relies on a comprehensive evaluation of clinical manifestations, laboratory tests, and imaging studies (10). However, clinical manifestations and laboratory tests lack specificity, making the integration of imaging studies particularly important. However, most clinical doctors lack systematic training in imaging, therefore heavily relying on imaging reports prepared by radiologists. In actual practice, due to various factors such as professional expertise and experience, different radiologists may provide different reports for the same patient's chest and abdomen films, which can affect the treatment decisions of clinical doctors and ultimately the health and development of the infants. Early and rapid diagnosis of NEC has always been a major challenge in intensive care clinical practice.

Multimodal deep learning models have attracted increasing attention in the field of artificial intelligence in recent years (11–14). Different forms or sources of information can all be referred to as modalities, and data composed of two or more modalities are called multimodal data. As a large amount of data of various types is generated in clinical practice, multimodal deep learning models have been widely applied and developed in the medical field. In the field of NEC-assisted diagnosis, the published studies mainly focus on the use of single-modal data such as laboratory test indicators or abdominal x-rays and ultrasound for NEC recognition and assisted diagnosis (15–18). However, these models have poor generalization, and the changes in NEC patients' blood parameters and imaging data have not been fully explored.

In order to achieve early, rapid, economical, and standardized diagnosis of NEC in clinical scenarios with imbalanced medical resources, in this study, we constructed an AI-based multimodal model and used admission laboratory test data and abdominal x-ray image data to build, train, and evaluate the model. Then, we conducted interpretability analysis and external validation with clinical doctor data, achieving the best-known performance in assisted diagnosis. This effectively assists clinical doctors in diagnosing and treating NEC in underdeveloped areas, increasing the early diagnosis rate of NEC, reducing misdiagnosis, missed diagnosis, and the occurrence of complications, and protecting the health of children.

## Method

### Dataset establishment

**Figure 1:**
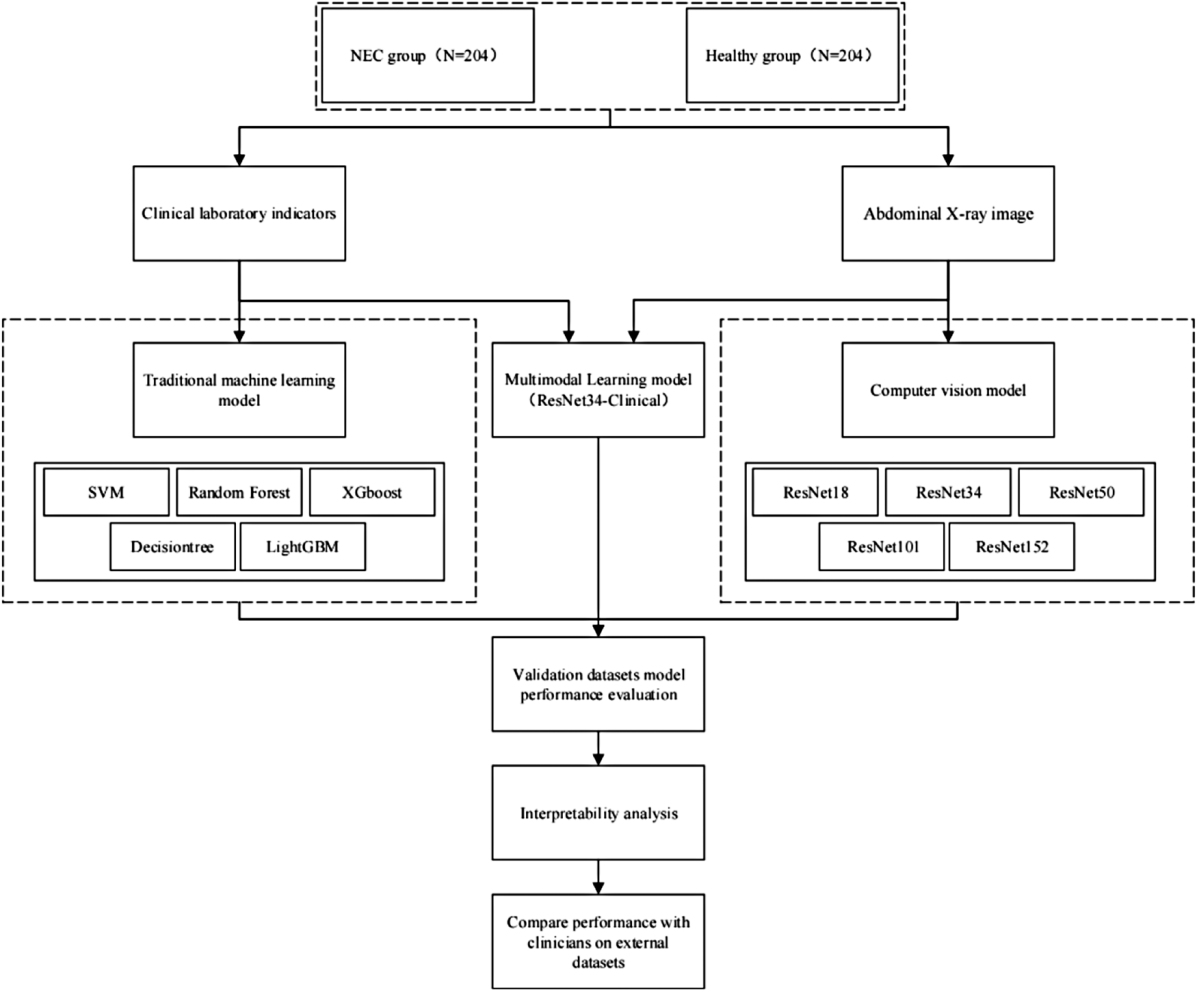
Study design flowchart.

This study is a cohort study. A total of 408 newborns who were admitted to our NICU from January 2022 to January 2024 were selected as the research subjects, including 204 cases of NEC patients and 204 cases of healthy infants. All NEC patients met the diagnostic criteria for NEC. The diagnostic criteria are based on the 2020 NEC diagnostic guidelines. Figure 1 shows the flowchart of this study.

The initial blood laboratory examination indicators (12 laboratory indicators determined according to the NEC diagnosis and treatment guidelines and clinical experience of doctors) and abdominal x-ray images of the research subjects were collected as research data. Inclusion criteria for image collection: (1) The patient is diagnosed with NEC for the first time. (2) Complete laboratory examination and abdominal x-ray examination are performed before treatment. Exclusion criteria: (1) Conditions such as pneumonia, congenital heart disease, sedative use, etc., may affect the quality of abdominal x-ray image diagnosis. (2) Non-first admission children. (3) Missing laboratory test indicators exceeding 30%. (4) The presence of conditions such as pneumonia, septicemia, etc., which may affect the diagnosis of laboratory test results.

All participants signed informed consent forms. This study was approved by the Hospital Ethics Review Committee. All data were strictly protected. All methods comply with relevant guidelines and regulations.

### Data processing

A total of 408 newborns were included in this study, including 204 non-NEC newborns and 204 newborns diagnosed with NEC, gestational age ranging from 26 to 40 weeks, and an average gestational age of 33 weeks. A total of 11,016 laboratory test data of the 408 children and 408 abdominal x-ray images were analyzed. The dataset was partitioned into training and validation sets in an 8:2 ratio, comprising laboratory assay results and abdominal x-ray images from 326 to 82 children, respectively. The distribution of demographic data is shown in Table 1.

**Table 1:** Comparison of clinical characteristics between the normal group and the NEC group in the cohort.

Clinical characteristic	Non-NEC (*N* = 204)	NEC (*N* = 204)	Total (*N* = 408)
Sex			
Male	100	98	198
Female	104	106	210
Gestational age			
<30	82	84	166
30–34	62	65	127
35–37	41	40	81
38–40	19	15	34

According to the NEC diagnosis and treatment guidelines, 27 laboratory examination data were included. These parameters include hematology analysis: neutrophil count, platelet count, lymphocyte count, neutrophil percentage, neutrophil-lymphocyte ratio, platelet-lymphocyte ratio, white blood cell count, and hemoglobin. In addition, C-reactive protein (CRP), procalcitonin (PCT), interleukin-6 (IL-6), and blood gas analysis, etc., were included.

Abdominal x-ray image data were collected from newborns who met the inclusion criteria, and x-ray examinations were performed using the Carestream DRX Revolution machine. The reason for using abdominal x-ray examination in this study is that abdominal ultrasound requires a professional ultrasonographer to achieve good results, and it is highly subjective. Abdominal x-ray examination has higher stability, can reflect the progression of NEC, and can be performed in hospitals at all levels. It has good generalizability and is also a reliable evidence recommended in clinical guidelines. In order to reduce the overfitting phenomenon during model training, image enhancement techniques such as horizontal flipping, rotation, and stretching were used to enhance the x-ray images in the dataset. All images were downsampled and converted into JPG images with a resolution of 256 × 256.

### Model design

This paper proposes a multimodal classification method based on Residual Neural Network (ResNet) (19) for processing images and one-dimensional CNN for processing data. By fusing the features of images and one-dimensional data in the fully connected layer, the joint classification of multimodal data is achieved. The residual network addresses the optimization training difficulties of neural networks when the depth increases by introducing residual blocks. The laboratory test results are processed by one-dimensional CNN to construct a multimodal network that simultaneously processes laboratory and symptom images.

To evaluate the performance of the multimodal model, five single-modal residual convolutional networks (ResNet18, ResNet34, ResNet50, ResNet101, ResNet152) and separate traditional machine learning models [Support Vector Machines (SVM), Random Forest, Decision Tree, XGBoost, and LightGBM] were also designed for model training and evaluation.ResNet (Residual Network) is a type of deep neural network architecture characterized by the introduction of residual connections, allowing the network to more easily learn identity mappings during training and alleviating the problem of vanishing or exploding gradients. Traditional machine learning models typically refer to models based on statistical learning theory, which are often composed of predefined mathematical functions. The parameters of these models are adjusted through training data to minimize predefined loss functions. Deep neural networks like ResNet often have deeper hierarchical structures and are capable of solving complex tasks by learning richer feature representations, whereas traditional machine learning models tend to rely more on handcrafted features or shallow feature extractors to address relatively simple tasks. In order to reduce the training time cost of the model, we introduce the idea of transfer learning and select the ResNet34 model pre-trained on the ImageNet dataset as the benchmark model for preliminary training, and perform multi-modal model training through local unfreezing. Ten-fold cross-validation was used for validation. Adam optimizer and grid parameters were used for training. The parameters that generated the minimum loss function value in the validation dataset within 100 epochs were selected as the best-performing model.

The hyperparameters were set as Batch size 32 and learning rate 0.000001. The random seed was set as 1024. The model training, construction, and validation were performed using Pytorch (2.2.0) (20) on a computer with an AMD EPYC 7532 processor (32 cores, 64 threads, 2.4–3.3 GHz) and 4×RTX 4090 cards (24GB GDDR6X VRAM, 16,384 CUDA cores).

### Model validation

In this study, the multimodal model was combined with gradient-weighted class activation mapping (GradCAM) (21, 22) for attention analysis, enhancing the interpretability of the model and the confidence of doctors. Global average pooling was applied to the last convolutional layer of the trained AI model using a classification activation map. The training weights of each output of the global average pooling layer indicate the importance of each feature map from the last convolutional layer. Then, weights were applied to the corresponding feature maps to generate significance maps. These significance maps were overlaid on the original abdominal x-ray images to visually differentiate the regions of interest prioritized by the multimodal model. To assess the model's performance, we additionally collected an external validation cohort consisting of 50 pediatric cases for prospective validation. After the diagnosis team made a definitive diagnosis, a human-machine comparison test was conducted: three doctors with advanced professional titles in neonatal ICU (unaware of the diagnosis results) and the model were asked to assess the cases based solely on laboratory tests and abdominal x-ray images.

### Statistical analysis

Performance indicators, including accuracy, sensitivity, specificity, area under the curve (AUC), and F1 score, were compared between the proposed model and existing methods. All analyses were conducted using R version 4.0.0.

## Results

### Demographic data

A total of 408 newborns were included in this study, including 204 non-NEC newborns and 204 newborns diagnosed with NEC, gestational age ranging from 26 to 40 weeks, and an average gestational age of 33 weeks. A total of 11,016 laboratory test data of the 408 children and 408 abdominal x-ray images were analyzed. The distribution of demographic data is shown in Table 1.

In order to improve the efficiency of model training and reduce the interference of irrelevant laboratory test results on model training, we conducted significance analysis on all laboratory test results (blood routine, biochemistry, immunity, etc.). The results after screening are shown in Table 2.

**Table 2:** Laboratory test items included in the normal group and NEC group in the dataset.

	Non-NEC (*n* = 204)	NEC (*n* = 204)	*P*
CRP(mg/L)	2.9	12.1	<0.001**
PCT(ng/ml)	0.7	2.34	<0.001**
Neutrophil percentage (%)	52	45	<0.001**
Platelets(10^9^/L)	152	132	<0.001**
Interleukin 6 (pg/ml)	12	16	<0.001**
Leukocyte (10^9^/L)	11.4	9.5	<0.001**
Transferrin (g/L)	1.2	0.7	<0.001**
Total protein (g/L)	49.6	59.1	<0.001**

### Model evaluation

**Figure 2:**
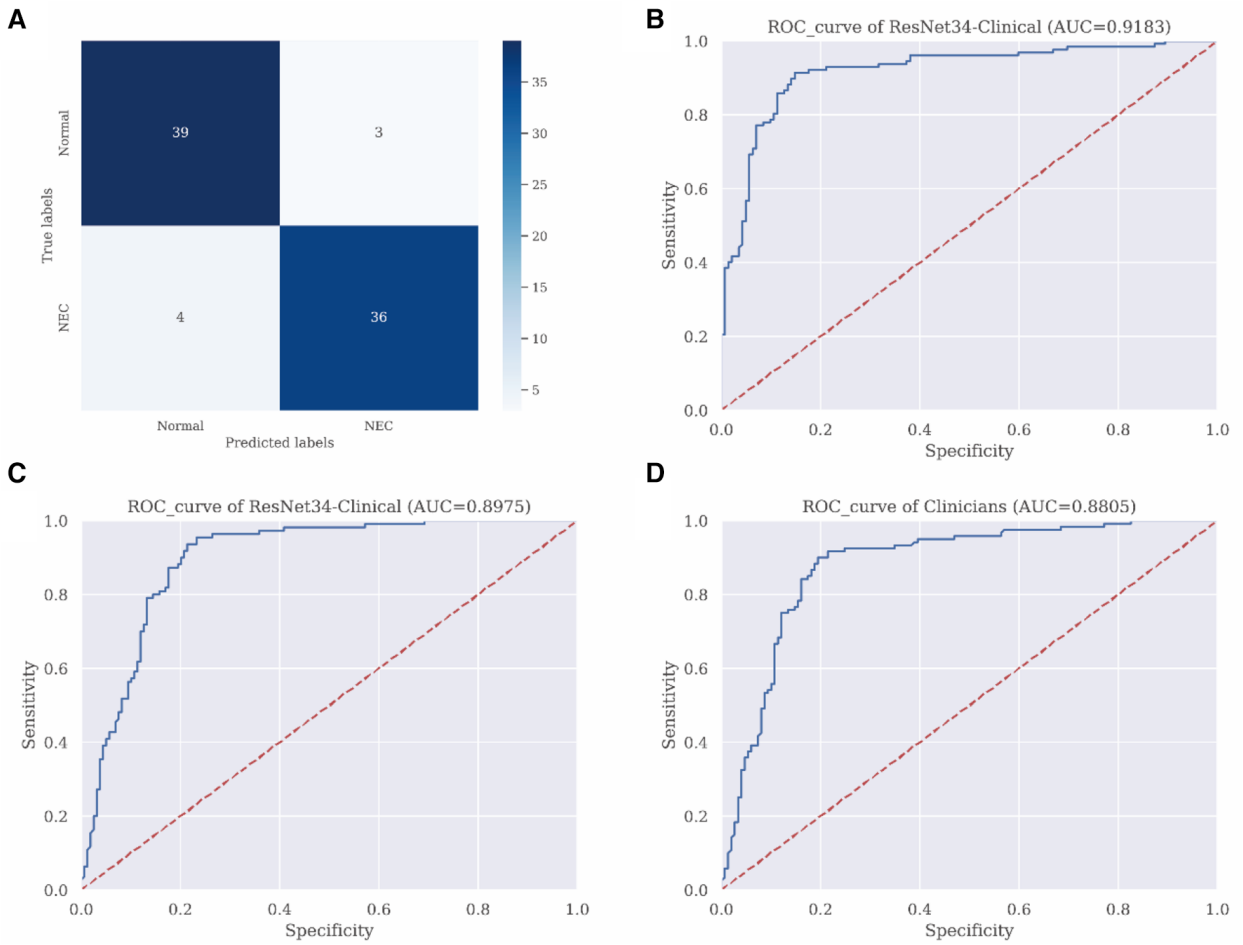
Performance evaluation of ResNet34-clinical model. (**A**) Confusion matrix of ResNet34-Clinical on the validation dataset. (**B**) Receiver operating characteristic curves of the ResNet34-Clinical model on the validation dataset. (**C**) Receiver operating characteristic curves of the ResNet34-Clinical model on the external independent dataset. (**D**) Receiver operating characteristic curves of clinical doctors on the external independent dataset.

In this study, ten-fold cross-validation was used to evaluate the performance of each model, with the validation dataset being used for this purpose. Each data point included laboratory test results and abdominal x-ray image data of a newborn. Different types of data were used based on the structure of different models. Table 3 displays the diagnostic performance of each model using the validation dataset. Traditional machine learning models (SVM, RF, XGboost, Decisiontree, LightGBM) had diagnostic accuracies of 79.78%, 80.32%, 77.41%, 81.94%, and 80.51% respectively, significantly lower than the computer vision model (ResNet). The trained multimodal deep learning model (ResNet34-Clinical) exhibited the best performance in terms of accuracy, sensitivity, specificity, and F1 score, significantly outperforming both traditional machine learning models and single computer vision models. The confusion matrix of the ResNet34-Clinical model can be seen in Figure 2A.

**Table 3:** Diagnostic performance of deep learning algorithms. The best performance is highlighted in bold, while the second best performance is underlined.

Model	Accuracy	Sensitivity	Specificity	Precision	F1 score
SVM	0.7978	0.7915	0.7927	0.8049	0.8172
RF	0.8032	0.7811	0.8188	0.759	0.8194
XGboost	0.7741	0.8034	0.8192	0.8083	0.7746
Decisiontree	0.8194	0.8056	0.8139	0.8053	0.7956
LightGBM	0.8051	0.7523	0.7949	0.7543	0.7612
ResNet18	0.8644	0.8949	0.8234	0.8445	0.8554
ResNet34	0.9199	0.9145	0.8668	0.8606	0.8493
ResNet50	0.8564	0.8725	0.8366	0.8408	0.8646
ResNet101	0.821	0.8257	0.8395	0.9062	0.8449
ResNet152	0.8475	0.8134	0.8662	0.9064	0.8928
ResNet34-Clinical	0.942	0.9365	0.9228	0.9302	0.9557

A recall curve was plotted for the ResNet34-Clinical model, which achieved an AUC of 0.92 (Figure 2B). To assess the stability and generalizability of the model, we designed an additional external validation group of 50 cases for evaluation, comparing the model's ROC curve with the ROC curve of human clinicians. The diagnostic performance of the multimodal deep learning model (AUC = 0.83) was comparable to that of human clinicians (AUC = 0.82) (Figures 2C,D).

**Figure 3:**
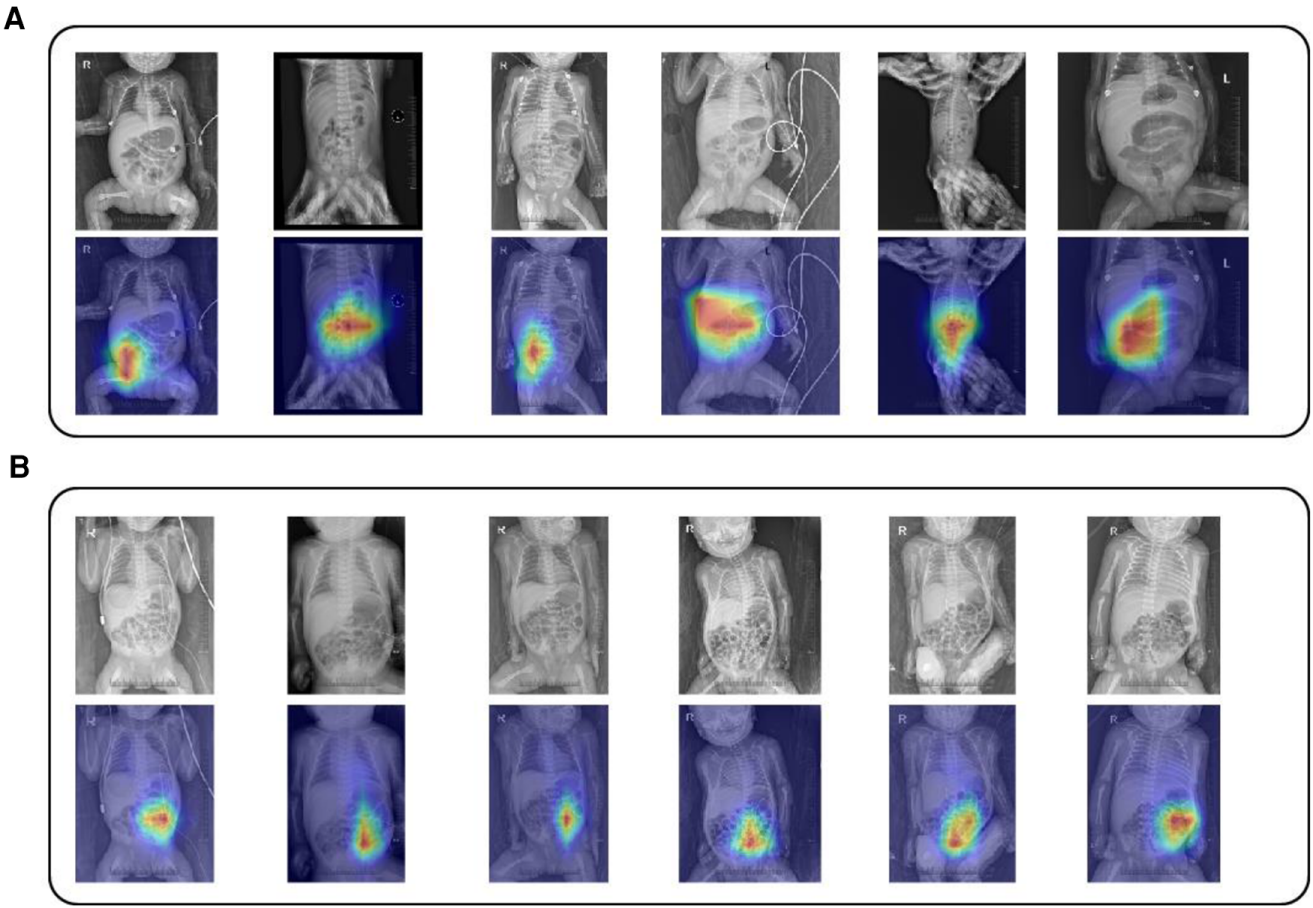
ResNet34-Clinical grad-CAM images (**A**) Overlaid images of abdominal x-rays and GradCAM for NEC patients. (**B**) Display of overlaid images of abdominal x-rays and GradCAM for non-NEC newborns. The thicker red area indicates the image portions that ResNet34-Clinical focuses on during the classification of NEC patients and healthy non-NEC newborns.

In order to enhance confidence and interpretability of the model in clinical scenarios, we applied the GradCAM technique to the model. After validating the abdominal x-ray images in the validation dataset, we found that the model's attention was mainly focused on fixed dilation of intestinal loops, intestinal wall edema, intussusception, and portal venous gas, which aligns with clinical experience and demonstrates the feasibility of our model. The evaluation of model attention can be seen in Figure 3.

## Discussion

This study developed an artificial intelligence-based multimodal model to assist clinical doctors in early diagnosis of NEC. Among the published multimodal studies, the best performance was achieved by WENJING GAO's ResNet50 model (17), a deeper neural network, trained on a large-scale dataset (*n* 3= 4,535), yielding more accurate results (ROC = 0.93). In contrast, our study utilized a faster training speed, shallower neural network, ResNet34 model, achieving comparable results (ROC = 0.91) on a smaller dataset (*n* = 408), suggesting that lightweight models may have better applicability in medical image analysis due to limitations in data scale. To enhance clinical confidence in our model, we not only integrated the approach proposed by GAO, employing gradient-weighted class activation mapping to explain the model's decision process, but also conducted an additional human-machine comparative experiment with an external validation cohort, achieving promising results (ROC = 0.83). The visually significant regions identified by GradCAM are consistent with clinical experiences, including fixed dilation of intestinal loops, intestinal wall edema, intramural gas, and portal vein gas. Our multimodal model can aid clinical doctors in early and accurate diagnosis of NEC in medically underserved areas, reducing complications and even fatalities resulting from misdiagnosis.

Our multimodal model does not require special laboratory tests and can be routinely implemented for NEC diagnosis by integrating routine laboratory tests available in every hospital with standardized abdominal x-ray images. The performance of the multimodal model is significantly superior to that of other single-modal models and traditional machine learning models.

Limitations: Firstly, since this study is a single-center study, in order to ensure the generalizability of the model, we plan to validate and test the model with a larger dataset from multiple centers. Secondly, the performance of the model in this study is similar to the diagnosis accuracy of pediatric doctors with ten years of clinical experience. We plan to establish a larger-scale dataset in the next step to improve the accuracy of the multimodal model. Thirdly, for the diagnosis of NEC, abdominal ultrasound images are also crucial. Our multimodal model currently only processes laboratory results and abdominal x-ray image information. We plan to incorporate an abdominal ultrasound data processing module in the next step to fully exploit clinical data and further improve the model's performance.

In this study, we developed a multimodal model using deep learning that can assist clinical doctors in early and rapid diagnosis of NEC. This model has broad prospects for assisting in the diagnosis of NEC in medically underdeveloped areas, enabling doctors to achieve early, convenient, economical, and accurate diagnosis and treatment of NEC, better safeguarding the health of newborns.

## Data Availability

The raw data supporting the conclusions of this article will be made available by the authors, without undue reservation.
